# Liquid biopsy in colon cancer: comparison of different circulating DNA extraction systems following absolute quantification of *KRAS* mutations using Intplex allele-specific PCR

**DOI:** 10.18632/oncotarget.21134

**Published:** 2017-09-21

**Authors:** Vera Kloten, Nadine Rüchel, Nadina Ortiz Brüchle, Janina Gasthaus, Nils Freudenmacher, Florian Steib, Jolein Mijnes, Julian Eschenbruch, Marcel Binnebösel, Ruth Knüchel, Edgar Dahl

**Affiliations:** ^1^ Molecular Oncology Group, Institute of Pathology, University Hospital Aachen, Aachen, Germany; ^2^ Centralized Biomaterial Bank of RWTH Aachen University (RWTH cBMB), Institute of Pathology, University Hospital Aachen, Aachen, Germany; ^3^ Department of Visceral and Transplantation Surgery, University Hospital Aachen, Aachen, Germany

**Keywords:** liquid biopsy, KRAS, cfDNA extraction systems, ctDNA quantification, intplex-allele specific PCR

## Abstract

Non-invasive molecular analysis of circulating tumor DNA (ctDNA) is a promising application in personalized cancer management, although there is still much to learn about the biological characteristics of ctDNA. The present study compared absolute amounts of *KRAS* mutated ctDNA and total circulating cell-free DNA (cfDNA) in colorectal cancer (CRC) patients (n=50) from various stages and healthy controls (n=8) by Intplex allele-specific and digital droplet PCR. In addition, the impact of two prominent extraction techniques (silica-based membrane vs. magnetic beads) on cfDNA and ctDNA recovery was analyzed in 38 paired samples from CRC patients and specific spike-in DNA controls. CfDNA fragment size was assessed using the Agilent 2100 Bioanalyzer. Relative quantities of total cfDNA quantities were measured using the Qubit fluorometer. Statistical analysis on total cfDNA yield revealed a strong correlation (r=0.976) between Qubit and absolute Intplex allele-specific PCR measurements in cancer patients and healthy controls. Total cfDNA was significantly increased in cancer patients compared to healthy controls, with the highest yield in distant metastatic disease. In line, the highest amount of ctDNA (1.35 ng/μL) was found in patients with distant organ metastasis. Of great interest, the silica-based membrane method significantly promoted extraction of long cfDNA fragments. In contrast, the magnetic bead system more efficiently recovered short cfDNA fragments in serum of cancer patients. Further, a decreased *KRAS* allele frequency was observed in serum compared to plasma. This study suggests that the source of cfDNA and choice of pre-analytical extraction systems needs to be more carefully validated in routine clinical practice.

## INTRODUCTION

Blood-based molecular analysis of tumor-derived circulating DNA (ctDNA) is becoming an established tool for monitoring tumor burden and detection of resistance early during targeted cancer therapies [1–3]. In contrast to total free circulating DNA (cfDNA) levels, i.e. circulating DNA from different cells-of-origin like healthy, malignant and tumor microenvironmental cells, quantity of ctDNA harboring cancer-associated aberrations is stage- and entity-dependent [4]. In addition, Bettegowda and colleagues already revealed an extreme variability of mutated ctDNA fragments in e.g. colon cancer patients (between 1 and 100.000 mutated fragments) illustrating the need for highly sensitive detection techniques. Innovative technologies like digital and allele-specific PCR were shown to detect oncologic point mutations (e.g. *EGFR* or *KRAS)* with a sensitivity of 0.01%, i.e. 2 mutant allele copies in a background of 20.000 wild type alleles [5, 6]. However, recent studies investigating the secondary *EGFR* T790M resistance mutation in advanced non-small cell lung cancer (NSCLC) patients showed that about 20-30% of tissue T790M mutations were not detectable in plasma-ctDNA, even with highly sensitive digital PCR technologies [7]. While ctDNA-based detection of the *EGFR* T790M mutation is already an approved diagnostic application in those cases where no tissue biopsy is available, it becomes clear that recent developments in liquid biopsy are rather based on innovative technologies than on an established knowledge about the analytes themselves.

The variability of mutated ctDNA fragments in cancer patients provides evidence that the biology of different cancer (sub-) types may be a limiting factor for detecting ctDNA mutations. Thus, a better knowledge on size and fragmentation are necessary for reliable quantification and analysis on circulating DNA. Various studies on circulating DNA illustrated a characteristic apoptotic ladder pattern of 160 - 180 bp or multiples thereof (oligonucleosomes) in cancer patients [8]. Apoptotic fragmentation of circulating DNA results from a caspase-activated DNase CUTTING free linker DNA between the nucleosome core particle (∼ 146 bp), which varies from 10 - 80 bp in length depending on species and tissue type [8, 9]. In addition, fragmentation occurred by lysosomal DNase II after the dying cells are phagocytosed [10]. Recent studies also demonstrated an additional cleavage of 10 bp periodicity in relation to the helical pitch of nucleosome-bound cfDNA [11]. Contrary to the typical apoptotic size distribution, the work by Mouliere and colleagues [12, 13] described *KRAS* mutated ctDNA fragments from colorectal cancer patients which were mainly smaller than 145 – 160 bp as compared to circulating DNA from *KRAS* wild type patients and non-tumoral derived circulating DNA from healthy cells. In line, recent studies by Jiang [14], Snyder [11] and Underhill [15] et al. indicated a shift to smaller fragments in cancer patients. However, the dominant structure of circulating DNA was shown at 167 bp. With respect to pre-analytic variables the choice of cfDNA extraction systems could influence detection of oligonucleosomes recently demonstrated by Pérez-Barrios and colleagues [16]. Furthermore, different extraction systems demonstrated variable cfDNA yields [17], showing the importance of pre-analytical studies to improve our knowledge of circulating DNA for routine clinical practice.

In this study, we assessed the impact of cfDNA and ctDNA yield on sensitive *KRAS* G12S and G12D detection in serum of CRC patients using promising Intplex allele-specific PCR (Intplex PCR). To go beyond previous pre-analytical efforts, we systematically compared an automated magnetic bead-based extraction system with a widely used membrane-based method showing a significant increased extraction of high molecular weight cfDNA fragments using the membrane-based kit in serum of colorectal cancer patients.

## RESULTS

### Intplex PCR reveals an enhanced cfDNA quantity in serum of advanced CRC patients

Today, the use of plasma instead of serum is recommended by many laboratories since the abundant cfDNA yield in serum, probably due to the enhanced lysis of circulating lymphocytes during serum preparation, reduces the relative amount of ctDNA. However, keeping in mind that clinically highly valuable patient collectives [18–22] are based on serum samples, we wondered if innovative Intplex PCR, first published by Thierry et al. [6], could be a reliable technology for sensitive analysis of oncological *KRAS* mutations in CRC patients’ serum samples as well. To this effect, we decided to investigate the most frequently observed *KRAS* mutation in colorectal cancer patients, i.e. G12D with a frequency of 33.5 – 34.4% among *KRAS* mutated colorectal cancers. In addition, we investigated G12S known to occur with low frequency of 4.9 – 5.7% in *KRAS* mutated colorectal cancers.

**Figure 1:**
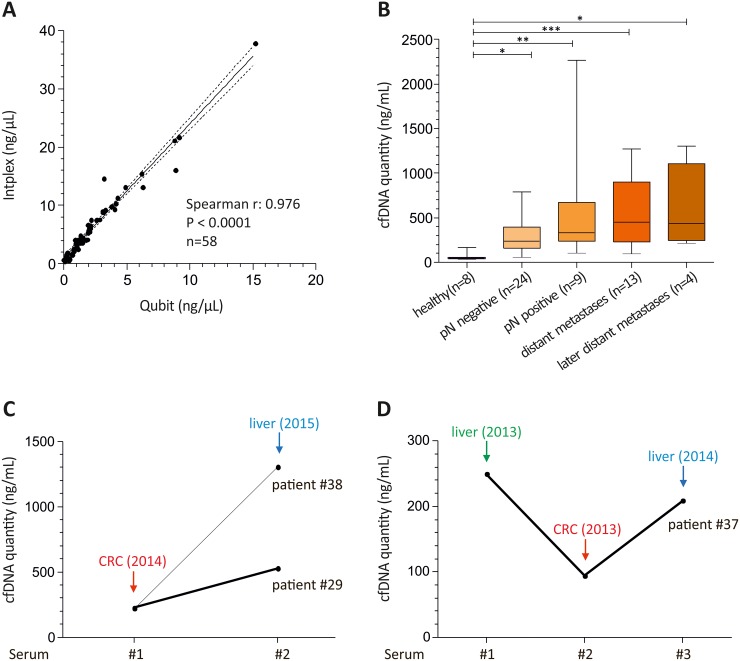
Intplex PCR shows abundant cfDNA levels in advanced colon cancer patients **(A)** Spearman correlation of serum cfDNA amount in eight healthy and 50 serum samples from CRC patients revealed a strong association between Qubit and independent Intplex PCR measurement. **(B)** Box plot analysis indicated significant higher cfDNA concentrations in CRC patients compared to healthy individuals. **(C)** and **(D)** In patients with advanced cancer and additional distant metastasis six months (patient #29 and #38) and 13 months (patient #37) after primary cancer diagnosis cfDNA concentrations were higher at the time point of second distant metastasis. In case of patient #37 the first blood sample was drawn before surgery of the liver metastasis (serum 1) and the second 24 days later before surgery of the colorectal tumor (serum 2). Serum 3 was drawn at diagnosis of the liver metastasis 13 months later. Statistical analysis was performed using 1way ANOVA Kruskal-Wallis test to compare all groups where; *P < 0.05, **P < 0.01, ***P < 0.001.

First, we compared serum cfDNA amounts in colon cancer patients (n=50) and healthy controls (n=8) measured with the Qubit system and the independent Intplex PCR revealing highly comparable results (spearman r: 0.976, P < 0.001) (Figure 1A). Further, absolute cfDNA quantification of CRC serum samples with Intplex PCR indicated a significantly increased cfDNA yield in CRC patients compared to healthy controls (median cfDNA: 49.5 ng/mL serum) (Figure 1B). In more detail, in contrast to healthy controls median cfDNA concentration was 4.7-fold, 6.7-fold and 9.1-fold higher in patients with lymph node negative (median cfDNA: 231.7 ng/mL serum), lymph node positive (median cfDNA: 330.1 ng/mL serum) and those with distant metastasis disease (median cfDNA: 450.8 ng/mL serum), respectively.

Of interest, metastasized CRC patients showed increased cfDNA concentrations at occurrence of a second distant metastasis diagnosed six months (n=2, patient #29 and #38) and 13 months (n=1, patient #37) after primary CRC (Figure 1C and 1D). In case of patient #37 the first blood sample was drawn before surgery of the liver metastasis (serum 1) and the second 24 days later, before surgery of the primary colorectal tumor (serum 2). Interestingly, cfDNA amount decreased 2.7-fold after the first surgery and reached a nearly identical level at diagnosis of liver metastasis 13 months later (serum 3) (Figure 1D).

### Intplex PCR detects *KRAS* mutations in serum of localized and advanced CRC patients

**Figure 2:**
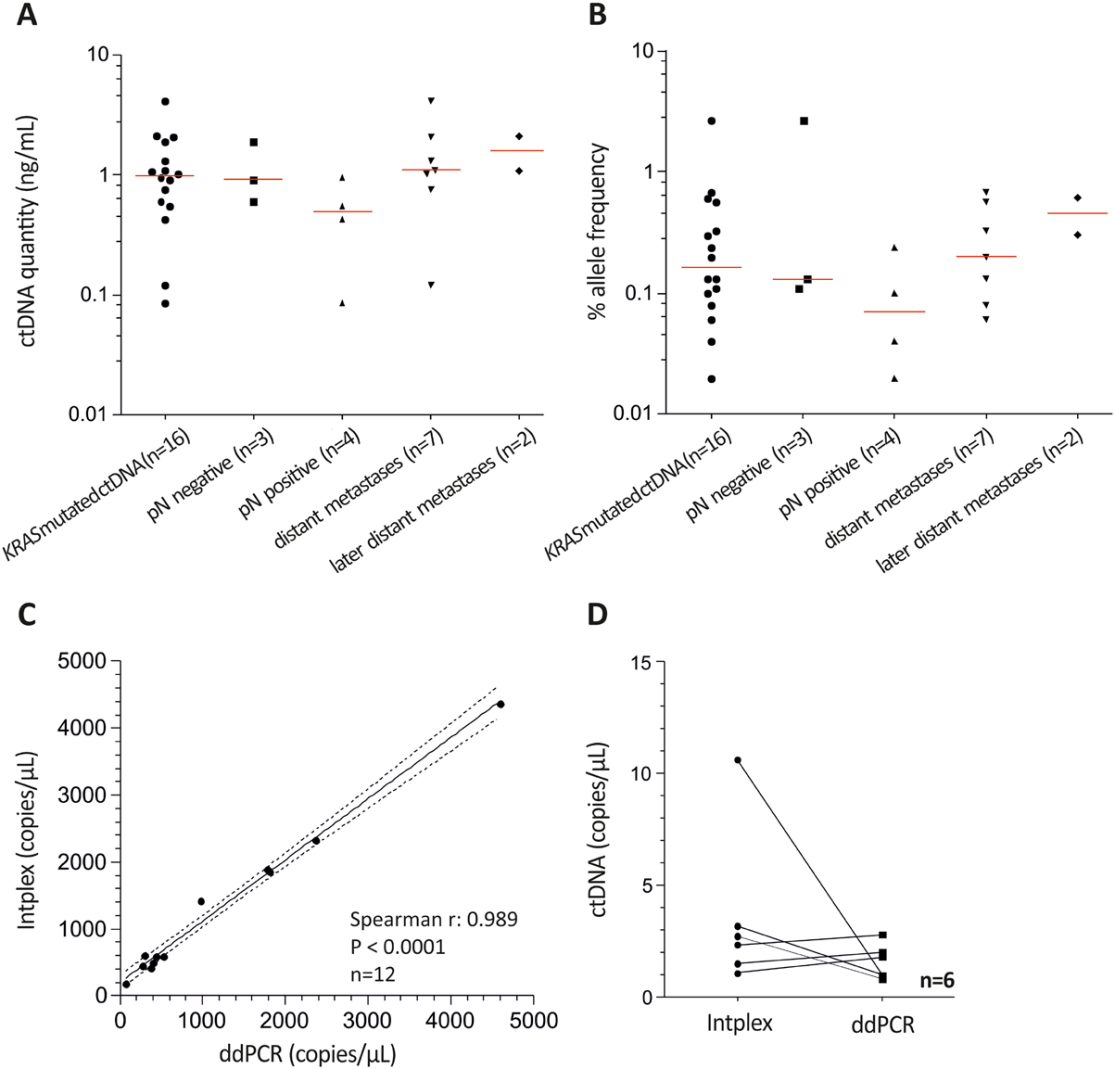
Intplex PCR indicates increased *KRAS* allelic frequency in serum of metastasized CRC patients **(A)** and **(B)** Scatter plot analysis indicated higher cfDNA concentrations and allelic frequencies in metastasized CRC compared to patients with local and lymph node positive healthy patients. Allelic frequencies ranging from 0.02% to 2.65% with the highest median value (0.46 %) in metastasized patients with a second diagnosed metastasis. **(C)** Spearman correlation of *KRAS* wild type allele copies revealed a strong association between ddPCR and independent Intplex PCR. **(D)** Measured *KRAS* G12D mutated allele copies were similar between Intplex and ddPCR.

Absolute quantification of *KRAS* G12D and G12S mutated ctDNA revealed a median concentration of 1.0 ng in 1 mL serum (Figure 2A). As expected, the highest amount of ctDNA was found in patients with distant organ metastasis. Allelic frequencies ranged from 0.02% to 2.65% with the highest median value (0.46%) in metastasized patients diagnosed with a second metastasis (Figure 2B). However, ctDNA quantity or allele frequency did not enable discrimination of different CRC stages.

Overall, we identified 16 patients (34.8%) who harbored either circulating mutant *KRAS* G12D (n=11; 23.9%) or G12S (n=3; 6.5%) or both (n=1; 2.2%). In more detail, 12.5% (3 of 24 serum samples were mutated) of patients with localized tumors revealed a *KRAS* mutation, while patients with lymph node positivity showed a *KRAS* mutation frequency of 44.0% (3 of 9 serum samples were *KRAS* G12D and one sample was G12S mutated). A *KRAS* mutation frequency of 53% (9 of 17 serum samples were mutated) was obtained in patients showing distant organ metastasis. Further, patient #37 showed a G12D mutation at diagnosis of the first liver metastasis (see Figure 1D, serum 1) and at diagnosis of an additional liver metastasis 13 months after primary CRC diagnosis (see Figure 1D, serum 3). Interestingly, a *KRAS* G12D mutation was non-detectable in serum 2 (see Figure 1D) after surgery of the first liver metastasis. In addition, patient #29 revealed a G12S mutation at diagnosis of liver metastasis (see Figure 1C).

We further investigated whether the obtained *KRAS* mutations in serum ctDNA were detectable in matched tissue DNA analyzed by Intplex PCR. Circulating *KRAS* mutations were not detected in 28 of 40 patients with *KRAS* wild type primary tumors, yielding a specificity of 70%. In addition, we identified five cases (of 10) in which mutations were present in the primary tumor tissue but not in serum ctDNA, yielding a sensitivity of 50%. Concordance between *KRAS* mutation status in the serum and tumor tissue was 66%. To strengthen Intplex PCR analysis, we investigated patients showing a *KRAS* G12D mutation in serum and/or tissue DNA by digital droplet PCR (ddPCR). Correlation analysis revealed a strong association (spearman r: 0.989, P < 0.001) of wild type *KRAS* allele copies measured by Intplex and ddPCR (Figure 2C). However, only 50% (6 of 12 of *KRAS* G12D cases) was confirmed with ddPCR with similar copy numbers (Figure 2D and Supplementary Figure 1).

### The use of the silica-based membrane system promotes the extraction of high molecular weight cfDNA fragments

Examined ctDNA concentrations and allelic frequencies were low. Therefore, we performed a more in-depth investigation of pre-analytical technologies. Since most of liquid biopsy studies use silica membrane-based systems, in many cases in the presence of carrier RNA, we wondered whether carrier RNA may be of influence on cfDNA yield and sensitivity of *KRAS* detection. A total of 38 serum samples from 10 CRC patients (all stages) were subjected to cfDNA extraction using two independent systems, both demonstrating successful extraction of cfDNA (Supplementary Figure 2). To understand whether the use of carrier RNA may influence cfDNA yield and/or absolute quantification of ctDNA with Intplex PCR we performed cfDNA extraction with and without the addition of 1 μg carrier RNA of paired samples. Our data indicate no significant difference in absolute cfDNA and ctDNA yield depending on the addition of carrier RNA or extraction technology (Supplementary Figure 2A and B).

**Figure 3:**
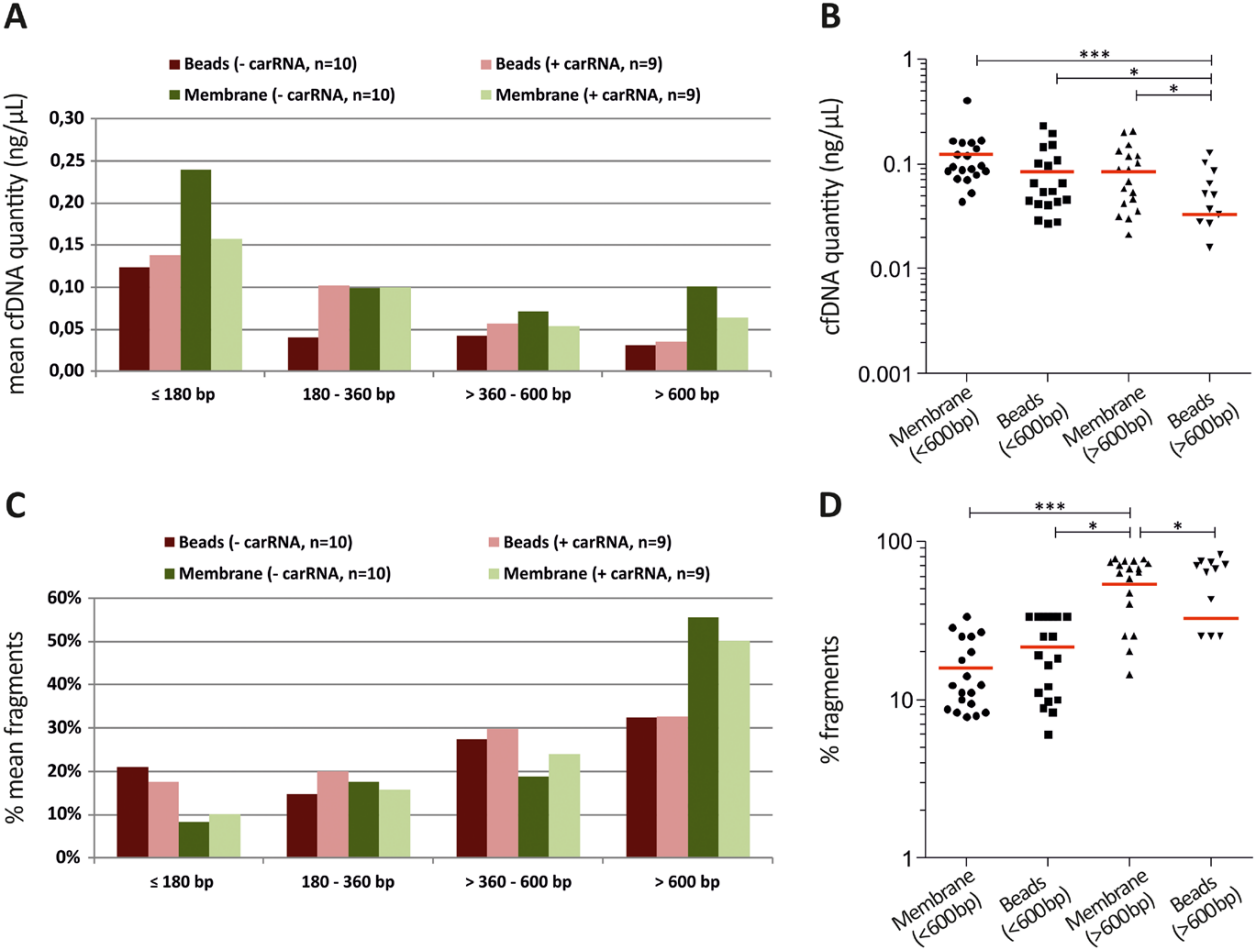
The use of the silica-based membrane system promotes the extraction of high molecular weight cfDNA fragments **(A)** The *silica-based membrane system* revealed increased cfDNA yields mainly of small (≤ 180 bp) and high (> 600 bp) fragments compared to the *magnetic beads system*. **(B)** Scatter plot analysis revealed a significant 2.5-fold increased concentration of high molecular cfDNA fragments (> 600 bp) using the *silica-based membrane system.* In line, significant increased amounts of small cfDNA fragments (< 600 bp) compared to high molecular cfDNA fragments (> 600 bp) presented only with the *magnetic beads system*. **(C)** The *silica-based membrane system* showed reduced percentage (normalized to total measured fragments) of small cfDNA fragments compared to the *magnetic beads system.* **(D)** Scatter plot analysis indicated a significantly increased percentage of > 600 bp cfDNA fragments using the *silica-based membrane system.* Histograms in (A) and (C) present mean values of n=18 matched samples (extracted with carrier RNA) and n=20 matched samples (extracted without carrier RNA) from 10 CRC patients categorised in distinct fragment size groups. Red horizontal line in (B) and (D) shows mean of the mentioned groups. Statistical analysis was performed using 1way ANOVA Kruskal-Wallis test to compare all groups where; *P < 0.05, ***P < 0.001.

To go beyond cfDNA quantification with the Qubit system or Intplex PCR we additionally performed a cfDNA fragment analysis using Agilent’s *2100 Bioanalyzer* system. The *silica-based membrane system* showed a slightly increased quantity of low sized cfDNA fragments (< 600 bp) compared to the *magnetic beads system*. However, only the *magnetic beads system* revealed significant increased amounts of small cfDNA fragments compared to high molecular cfDNA fragments (> 600 bp). Interestingly, the *silica-based membrane system* displayed a significant 2.5-fold higher concentration of high molecular cfDNA fragments (> 600 bp) (Figure 3A and 3B). In line, the *silica-based membrane system* showed a significantly elevated percentage (normalized to total measured fragments) of > 600 bp cfDNA fragments (Figure 3C and 3D), which are characteristic for cfDNA originating from lysed lymphocytes during blood processing. In contrast, the *magnetic beads system* showed a 1.6-fold increased percentage of extracted low sized cfDNA fragments compared to *silica-based membrane system* (Figure 3C and 3D).

### The use of serum leads to a lower allelic frequency of *KRAS* mutations compared to plasma

Showing an increased yield of high molecular serum cfDNA fragments extracted using the *silica-based membrane system* we wondered if there is an influence on the detection of *KRAS* mutations depending on cfDNA-source. To shed light on this question, we next evaluated ctDNA and allelic frequency coverage using both extraction systems in plasma and serum samples from healthy individuals using spike-in DNA harboring a clinically relevant *KRAS* G12D or G12S. As expected, absolute amounts of serum-cfDNA were higher compared to plasma-cfDNA levels: Based on two independent spike-in experiments a 6.7-fold higher median cfDNA concentration was shown for the *magnetic beads system*, while the *silica-based membrane system* demonstrated 3.7-fold increased cfDNA yield in serum compared to plasma samples (Supplementary Figure 3).

**Figure 4:**
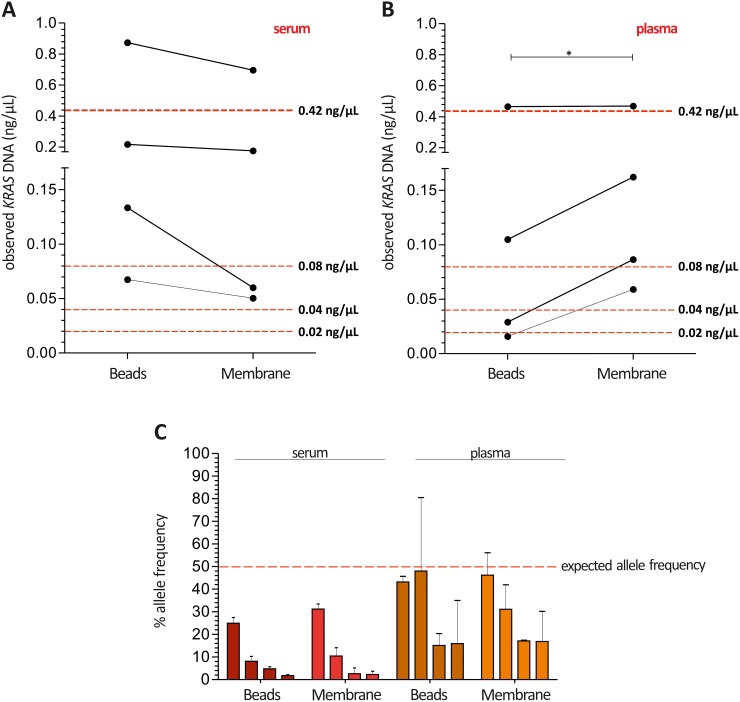
Higher cfDNA yields in serum may result in a lower allelic frequency of *KRAS* mutations compared to plasma Median *KRAS* G12D and G12S ctDNA concentrations measured in serum **(A)** were slightly higher as the expected concentrations, while absolute ctDNA quantification in plasma **(B)** represents the amounts of spike-in DNA with better accuracy showing the best suitable quantities for the *magnetic beads system* (red dotted line: expected ctDNA yield). **(C)** Allelic frequencies were much lower than expected in serum samples compared to plasma using Intplex PCR (red dotted line: expected allele frequency). Expected ctDNA yield calculated concerning the spike-in DNA concentrations 50 ng/μL – 10 ng/μL – 5 ng/μL – 2.5 ng/μL eluted in 60 μL elution buffer and 50% allele frequency. Statistical analysis was performed using a paired Student’s t-test where; *P < 0.05.

Next, mean G12D and G12S spike-in DNA concentrations measured in serum were slightly higher as expected (Figure 4A). In contrast, absolute quantification of plasma spike-in DNA revealed a more accurate resemblance using the *magnetic beads system*, while with the *silica-based membrane system* significant higher concentrations were retrieved (Figure 4B). In addition, neither the samples without spike-in DNA nor *KRAS* wild type spike-in DNA gave an unspecific signal in serum or plasma. Based on absolute cfDNA and ctDNA quantification we calculated the allelic frequency of the mentioned *KRAS* mutations, which is expected to be 50% in all samples. Probably due to the elevated cfDNA levels, allelic frequencies were much lower than expected in serum samples compared to plasma (Figure 4C).

## DISCUSSION

Today, molecular analysis of e.g. circulating tumor DNA in bodily fluids (also known as liquid biopsy) offers a supplementary option for accurate mutation testing in patients without available tumor tissue or to measure tumor burden early at disease relapse. Recent studies [2, 3, 6] showed the detection of clinically relevant oncologic driver mutations like e.g. *KRAS*, *EGFR* or secondary *EGFR* T790M resistance mutations in ctDNA with nearly maximal specificity (> 95%) compared to tumor tissue as the gold standard in cancer diagnostics. On the contrary, although digital PCR methods revealed a maximal technical detection limit of 0.01% (i.e. two mutated copies in a background of 20.000 wild type alleles), sensitivity of the mentioned mutations in ctDNA was unexpectedly low in some studies (ranging from 30 to 70%) [7, 23]. Especially in early localized cancers the amount of detectable ctDNA is low [4]. In line, in the current study, of five *KRAS* G12D mutations found in tissue DNA of local lymph node negative CRC patients, only one mutation was detectable in matched serum ctDNA by Intplex PCR. Further, detection of mutated ctDNA in 10 patients with matched wild type tumor tissue DNA might be explained by tumor evolution during therapy, since seven of these patients were diagnosed with advanced stage CRC. In addition, it is obvious that more mutations are detected from ctDNA as compared to tumor tissue due to temporal, and intra-, or inter-clonal heterogeneity [4] resulting in a moderate concordance. Of importance, ctDNA mutations in serum with paired wild type tissue DNA could be an early indicator for tumor relapse or non-detectable (micro-) metastasis, since recent studies showed the application of ctDNA in monitoring of colorectal [24] and breast [25] cancer patients.

Unfortunately, therapy- or clinical follow-up data of the analyzed patients were not available. However, only 50% of Intplex *KRAS* G12D cases confirmed with only ddPCR. In addition, two cases with an Intplex *KRAS* ctDNA wild type indicated as mutated with ddPCR. Keeping in mind that *KRAS* mutated ctDNA from colorectal cancer patients showed with a high quantity of low-size fragments (< 80 bp) [13] discrepancy of mutational status might be explained by different primer amplicon length. While Intplex PCR amplified a 60 bp sequence of the *KRAS* allele, ddPCR uses a 90 bp primer amplicon. However, a strong association (spearman r: 0.976, P < 0.001) of wild type *KRAS* allele copies measured with Intplex and ddPCR was found, underlining robustness and reliability of Intplex PCR.

Besides the biological ctDNA complexity the source of ctDNA, i.e. the use of serum in our study, could be a reason for lower sensitivity or discrepancy between Intplex and ddPCR. While many retrospective and clinically highly significant patient cohorts still rely on serum samples we addressed the reliability of the Intplex PCR technology in a comprehensive CRC serum collective. Comparing circulating mutational load of *KRAS* G12D and G12S in distant metastasized CRC patients in our study to the work by Thierry et al. [6] revealed a 5.2-fold and 48.7-fold decrease in median ctDNA yield and allelic frequency, respectively. As expected, total cfDNA was 7.6-fold higher in our serum-based study compared to plasma analysed by Thierry et al. probably resulting in a lower ctDNA yield and allele frequency of detected *KRAS* mutations in our study. In line, we demonstrated a higher median cfDNA concentration using the *magnetic beads* or *silica-based membrane system* in serum compared to plasma samples of healthy individuals. Further spike-in experiments clearly demonstrated that a higher cfDNA yield in serum decreased the measurement of ctDNA allele frequencies with Intplex PCR independent of the used extraction technology.

Today, some research studies have been seeking to improve the pre-analytical steps of ctDNA processing by using specialized cell free DNA blood stabilization tubes [17, 26, 27]. In addition, different extraction methods for the isolation of cfDNA from serum or plasma samples were compared demonstrating that the extraction method can considerably affect cfDNA quantity [16, 17, 28]. Similarly, the current study observed that both silica- and magnetic beads-based methods yielded sufficient cfDNA for downstream PCR applications, while the magnetic beads system (used in this study) is a simpler and more rapid automated method. Further, similar absolute cfDNA and ctDNA yields with both technologies and conditions (+ and – carrier RNA) were observed. Interestingly, the use of a *silica-based membrane system* enhances the extraction of long cfDNA rather than shorter ctDNA fragments, in serum of CRC patients. Notably, the *magnetic beads system* more efficiently recovers both short and fragmented cfDNA, probably representing circulating DNA from apoptotic tumor cells. This observation should be validated in more patients since it has been suggested that low molecular weight cfDNA fractions often harbor genetic aberrations indicative of tumor-derived DNA [13, 15]. Concerning fragment size analysis, one should be aware that the *2100 Bioanalyzer* from Agilent revealed a more accurate fragment size recovery compared to the *TapeStation* (Agilent) system (Supplementary Figure 4).

To this end, careful consideration remains necessary for optimal cfDNA extraction, fragment size analysis and specific ctDNA amplification in liquid biopsy. Intplex allele-specific PCR is a sensitive technology for the absolute measurement of total cfDNA as well as detection of oncological driver mutations even in serum samples while reducing cost and time of analysis. Further, we demonstrated that there are significant differences in high and low molecular weight cfDNA fractions recovery according to the methodologies evaluated. A limitation of this study is the small number of samples studied (n=38 paired samples from 10 CRC patients) while larger studies are needed to evaluate the impact of such differences on downstream PCR applications in routine clinical practice. Underlining recent investigations, plasma should be the analyte of choice in liquid biopsy analyses. However, using the best suited (pre-) analytical system clinically valuable retrospective serum cohorts can be investigated with clinical purpose as well.

## MATERIALS AND METHODS

### Patients

Subject of this study was a cohort of 50 colorectal cancer patients including FFPE tissues and serum samples. Hematoxylin and eosin-stained sections of FFPE tissues were prepared for assessment of the percentage of tumor cells by a pathologist. The analyzed patient cohort represents various stages of colon cancer tumorigenesis compromising lymph node negative and distant metastasis negative cases (n=24), lymph node positive and distant metastasis negative cases (n=10), lymph node negative or positive and distant metastasis positive cases (n=16) and the metastases tissues (n=26) from different organ sites at different time points (i.e. metastases tissue at primary CRC diagnosis and at later distant metastasis).

For ctDNA analysis matched serum samples were available from 46 patients at primary cancer diagnosis including four patients with an additional serum sample at diagnosis of subsequent metastasis. Biomaterial samples were provided by the RWTH centralized Biomaterial Bank Aachen (RWTH cBMB, Aachen, Germany) in accordance with the regulations of the biomaterial bank and the approval of the ethics committee of the medical faculty, RWTH Aachen. Eight serum samples from healthy individuals were included as controls. All patients gave written informed consent for retention and analysis of their tissue for research purposes (local ethical review board of the medical faculty of the RWTH Aachen, ref no. EK-206/09). An overview of patients’ characteristics is given in Table 1. Tissue and paired serum samples were collected between 2012 and 2015. Blood was drawn prior to tumor tissue removal.

**Table 1:** Histo-pathological patient characteristics

Categorisation	Patients (n^a^=50)	%
**Median age at diagnosis (range)**	73 years (43-89)	
**Gender**		
female	23	46%
male	27	54%
**Localisation**		
right-sided	15	30%
left-sided	31	62%
unknown	4	8%
**Histological grade**		
G2	40	80%
G3	6	12%
unknown	4	8%
**pT**		
pT 1-2	13	26%
pT 3-4	34	68%
unknown	3	6%
**pN**		
pN negative	28	56%
pN positive	19	38%
unknown	3	6%
**pM**		
pM positive	14	28%
pM unknown	36	52%
**Localisation distant metastases**		
liver	16	
kidney	2	
ovary	1	
peritoneum	1	
omentum majus	1	
colon	4	

### Sample processing

Blood samples (10 mL) from all study participants were obtained by venipuncture. All samples were processed at room temperature within 3 h from the time of blood extraction. Hemolyzed samples were discarded for further analysis. For serum preparation blood was collected in tubes containing a clot activator (S-Monovette, Order no. 02.1063, Sarstedt, Nümbrecht, Germany). According to manufacturer’s protocol samples were centrifuged at 2.000 x *g* for 10 minutes at room temperature. Plasma was prepared from whole blood collected in K3 EDTA tubes (S-Monovette, Order no. 02.1066.001, Sarstedt, Nümbrecht, Germany) at 2.000 x *g* for 10 minutes at room temperature according to manufacturer’s recommendation. The resulting supernatant (either serum or plasma) was carefully aspirated from the tube (in case of plasma without disturbing the buffy coat layer) and transferred in 1 mL aliquots into 1.5 mL tubes, and then centrifuged a second time at 16.000 x g for 10 minutes to remove cellular debris. Serum or plasma aliquots were then transferred to a new 1.5 mL tube and stored at -80°C until use. At the time when the cfDNA extraction was performed samples were thawed once on ice. No freeze-thaw cycles of analysed blood samples was done.

### DNA extraction from FFPE tissue

DNA was extracted from 5 x 10 μm freshly-cut FFPE tissue sections using the *QIAamp DNA Mini kit* (Qiagen, Hilden, Germany) according to manufacturer’s protocol. DNA was eluted in 200 μl of elution buffer.

### DNA extraction from plasma and serum

A comparison of two extraction kits was performed with 38 paired serum samples from 10 colon cancer patients (metastatic or not) as well as plasma or serum from healthy donors with distinct concentrations of spike-in DNA. The following kits were used: *Maxwell RSC ccfDNA Plasma* kit (Promega, Madison, USA; designated in the manuscript as *magnetic beads system*) and *QIAamp Free Circulating Nucleic Acid* kit (Qiagen, Hilden, Germany; designated in the manuscript as *silica-based membrane system*). Samples were processed according to the manufacturers’ protocols with slight modifications: Serum samples from paired CRC patients were isolated using the *magnetic beads system* or the *silica-based membrane system* either with (+ 1μg carrier RNA) or without the addition of carrier RNA (- carrier RNA).

For spike-in experiments DNA exhibiting a *KRAS* G12S or G12D mutation (Horizon Discovery, Cambridge, United Kingdom; Batch: 16118 (G12D) and 20815 (G12S)) with an allelic frequency of 50% was used. A *KRAS* wild type DNA (Horizon Discovery, Cambridge, United Kingdom; allelic frequency 100%; Batch: 17605) was used as control. Serum or plasma without spike-in DNA was used as an additional negative control. Besides the original DNA concentration (50 ng/μL), spike-in DNA was diluted with 10 mM Tris Buffer to a final concentration of 25 ng, 5 ng and 2.5 ng of which 1 μL was spiked into 1 mL serum or plasma from healthy donors. Spike-in DNA was extracted with the magnetic bead or the silica membrane system according to the manufacturers’ protocols (*magnetic beads system* without carrier RNA, *silica-based membrane system* with carrier RNA) following absolute DNA quantification by Intplex PCR.

In all cases, cfDNA was isolated using as starting volume 1 mL of serum or plasma, eluted in 60 μL of the supplied elution buffer and stored at -80°C until use.

### Agilent 2100 bioanalyzer

Fragment analysis was performed at the Genomics-facility of the Interdisciplinary Center for Clinical Research (IZKF) at the university hospital Aachen. The *High Sensitivity DNA Assay* on the 2100 Bioanalyzer enabled analysis of cfDNA fragments between 35 and 10.000 bp, according to manufacturer’s protocol.

### Intplex allele-specific PCR

Intplex allele-specific PCR was performed according to the protocol by Thierry et al. [6] with modifications: qPCR amplifications were carried out in triplicate in a reaction volume of 25 μL on an IQ5 instrument using iQ5 Optical system software 2.0 (Bio-Rad, Munich, Germany). The 25 μL qPCR mix was composed of 12.5 μL of *GoTaq* qPCR Master Mix (Promega), 0.75 μL of each amplification primer (0.3 pmol/μL), 9.25 μl of nucleic acid–free water (Promega) and 1 μL of extracted cfDNA. Primer sequences, thermal cycling, melting curves and data analysis were investigated as mentioned by Thierry et al. [6]. To ensure specific detection of the targeted mutation, each run contained positive and negative controls. Positive control DNA was extracted from cell lines SK-LU-1 (confirmed G12D mutation) and A546 (confirmed G12S mutation). Serial dilutions of genomic cell line DNA were used as a standard for absolute quantification. *KRAS* wild type DNA was used as a negative control and autoclaved water was used as a non-template control.

### Digital droplet PCR

The Bio-Rad QX200 System was used for digital droplet PCR (ddPCR). The ddPCR reaction mixture was loaded into the emulsification device and droplets were formed following the manufacturer's instructions. The contents were transferred to a 96-well reaction plate and sealed with a pre-heated Eppendorf 96-well heat sealer for 2 seconds. Total DNA was amplified separately in a C1000 Touch Thermal Cycler (Bio-Rad). The ddPCR™ probe assay *KRAS* p.G12D, Human (Unique Assay ID: dHsaCP2500596) was used for analysis. Each reaction consisted of a 20 μL solution containing 10 μL ddPCR Probe Supermix, 450 nM primers, 250 nM probe, and 1 μL template DNA with the following cycling conditions: 10 minutes at 95°C, 40 cycles each consisting of a 30 second denaturation at 94°C followed by a 55°C extension for 60 seconds, and a final 10 minutes at 98°C. After cycling droplets were analyzed immediately. Absolute quantities of mutant and wild type *KRAS* cfDNA copies were determined using the *QuantaSoft* software. Briefly, the system uses a 2-color detection system for the wild type (HEX) and mutant (FAM) alleles to count the number of droplets positive for each fluorophore. We considered samples as positive for mutant *KRAS* when 1 positive FAM droplet were identified above the threshold level.

### Statistical analysis

Statistical analyses were performed using SPSS 22.0 (SPSS, Chicago, IL) and GraphPad Prism 5.0 (GraphPad Software Inc., La Jolla, CA). Box Plot graphs are shown as follows: *Horizontal lines*: grouped medians. *Boxes*: 25-75% quartiles. *Vertical lines*: range, peak and minimum. The 1way ANOVA Kruskal-Wallis test was used to compare cfDNA concentrations between distinct conditions. Correlation analysis was performed by calculating a *Spearman* correlation coefficient. Differences were considered statistically significant if the two-sided p-values were equal or below 5% (≤0.05).

## SUPPLEMENTARY MATERIALS FIGURES



## Abbreviations

bp: base pairs
cfDNA: free circulating DNA
CRC: colorectal Cancer
ctDNA: circulating tumor DNA
ddPCR: digital droplet PCR
*EGFR*: Epidermal Growth Factor Receptor
*KRAS*: Kirsten rat sarcoma viral oncogene homolog
NSCLC: non-small cell lung cancer
PCR: polymerase chain reaction
