# Supplementary material for: Liquid biopsy in colon cancer: comparison of different circulating DNA extraction systems following absolute quantification of KRAS mutations using Intplex allele-specific PCR
Source: Oncotarget. 2017 Sep 21;8(49):86253–63. doi: 10.18632/oncotarget.21134 (PMC5689682; doi:10.18632/oncotarget.21134)
Supplement: Supplementary file 1 [file oncotarget-08-86253-s001.pdf]

Liquid biopsy in colon cancer: comparison of different circulating DNA extraction systems following absolute quantification of *KRAS* mutations using Intplex allele-specific PCR

SUPPLEMENTARY MATERIALS

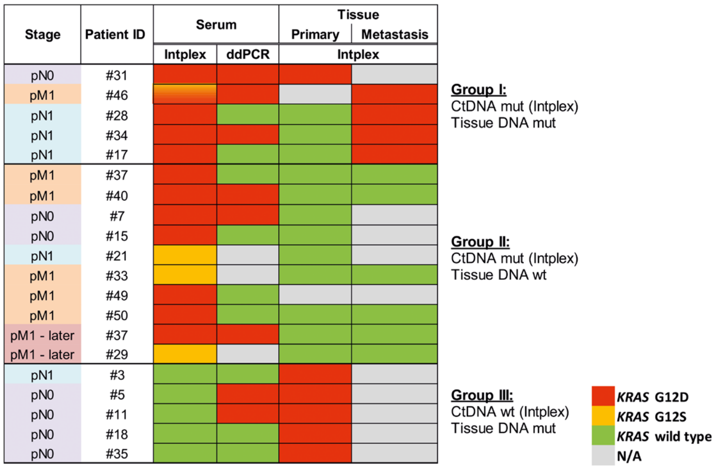

Supplementary Figure 1: Comparison of *KRAS* mutational status in tissue and serum DNA. Heatmap illustrates frequency of detected *KRAS* G12D and G12S mutations in serum ctDNA and matched tissue DNA. Cases were sorted according to the mutational status into three groups. Intplex, Intplex allele-specific PCR; ddPCR, digital droplet PCR; N/A, not available. Tissue samples with N/A are due to the lack of primary or distant metastasis tissue.

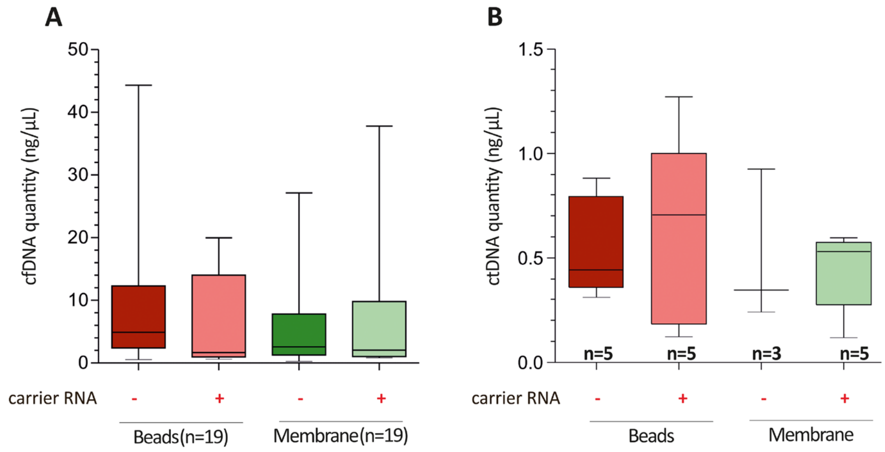

Supplementary Figure 2: Absolut amounts of cfDNA and ctDNA are similar between different extraction technologies and conditions. **(A)** and **(B)** Box plot analysis indicated no significant difference in absolute cfDNA and ctDNA yield according to the addition of carrier RNA or extraction technology. Statistical analysis was performed using 1way ANOVA Kruskal-Wallis test to compare all groups.

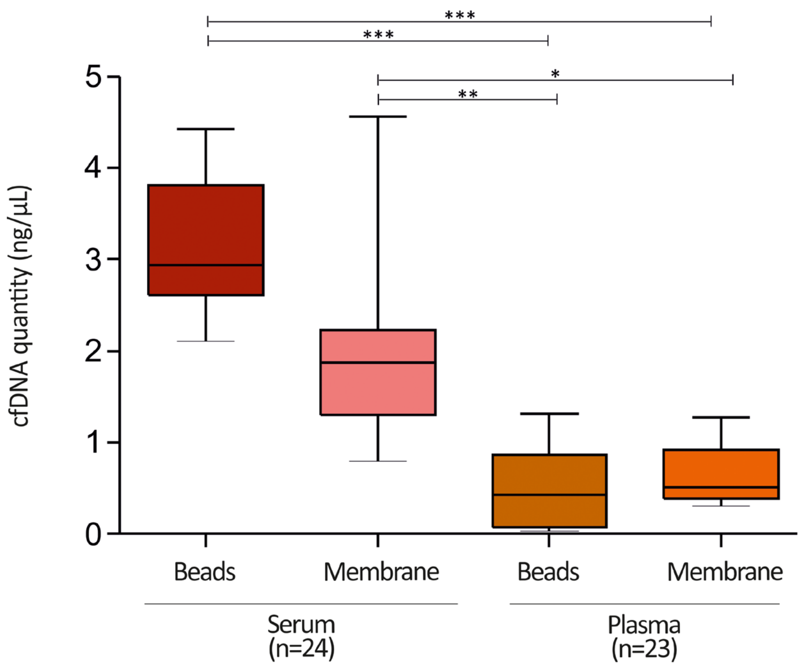

Supplementary Figure 3: Intplex PCR reveals increased cfDNA quantities in serum compared to plasma. Box Plot analysis revealed a higher median cfDNA concentration in serum of healthy individuals compared to plasma samples. Statistical analysis was performed using 1way ANOVA Kruskal-Wallis test to compare all groups where; $*P < 0.05$, $**P < 0.01$, $***P < 0.001$.

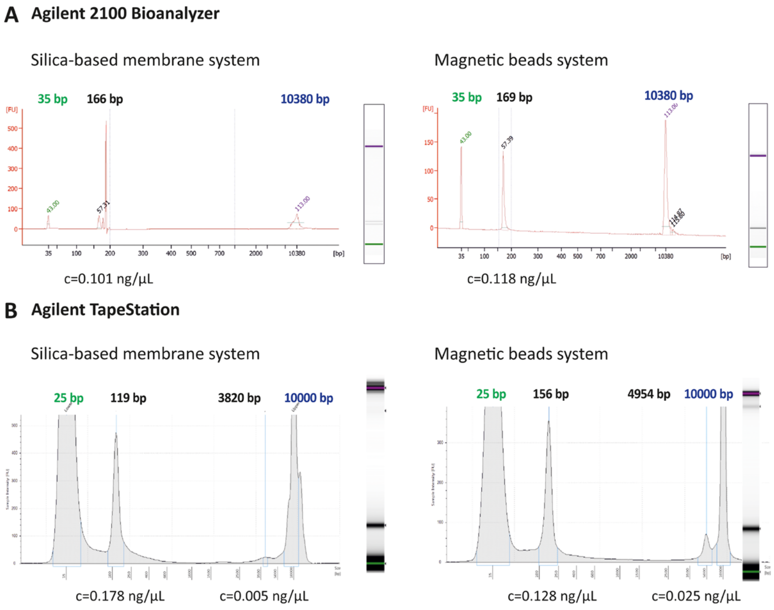

Supplementary Figure 4: The Agilent *2100 Bioanalyzer* shows an accurate fragment size recovery. A purified 170 bp PCR-product (10 ng) was spiked-in 1 mL of plasma from a healthy volunteer and eluted in 60 µL of the supplied elution buffer. DNA was extracted using either the *silica-based membrane system* (Qiagen) or the *magnetic beads system* (Promega) and fragment size was analysed using *Bioanalyzer* or *TapeStation*. The *Bioanalyzer* **(A)** recovered independent of the extraction technology the 170 bp spiked-in DNA fragment while the *TapeStation* **(B)** indicated a size shift of 51 bp using the *silica-based membrane system* and a size shift of 14 bp using the *magnetic beads system*. Extracted quantity of the 170 bp PCR-product was similar between different methods.
